# A new methodology for examining the efficacy of SBIRT protocols on reducing healthcare utilization and costs

**DOI:** 10.1186/1940-0640-10-S2-O44

**Published:** 2015-09-24

**Authors:** Janice Pringle, Arnie Aldridge, Shannon Kearney

**Affiliations:** 1 School of Pharmacy, Department of Pharmacy and Therapeutics, University of Pittsburgh, Pittsburgh, USA; 2 RTI International, Research Triangle Park, USA

## Background

There is increasing interest in deploying Screening, Brief Intervention, and Referral to Treatment (SBIRT) practices in emergency departments (ED). However, the current literature is inconclusive on whether or not SBIRT practices are cost effective and cost beneficial. In order to answer this question, new analytical methods need to be developed.

The objective of the following study was to pilot a new experimental methodology - modeling costs using a multilevel generalized linear model (GLM) - for studying the impact of SBIRT on healthcare utilization and costs.

## Material and methods

In the study, healthcare utilization and costs were quantified for patients who received emergency department (ED) services and participated in an SBIRT program entitled Safe Landing. The healthcare and utilization costs of patients who underwent the Safe Landing program were compared using a multilevel GLM to patients in three control groups where no SBIRT protocol was implemented.

## Results

The study found that SBIRT was associated with 24% lower health care costs from the 12 months preceding the index ED visit to the 12 months following the index ED visit. This translates to approximately $2,600 per patient per year. This reduction in healthcare costs could be linked mainly to decreased inpatient use. The study found a reduction in inpatient claims and visits, suggesting that the reduction in health care costs was driven mainly by a decrease in inpatient visits.

## Conclusions

This study provides further support that SBIRT programs are a cost-effective and cost-beneficial approach to substance use disorder management. The study also contributes to important literature on the impact of SBIRT implemented in real world settings, rather than traditional randomized clinical trials.

